# A web tool for the global identification of pig breeds

**DOI:** 10.1186/s12711-023-00788-0

**Published:** 2023-03-21

**Authors:** Jian Miao, Zitao Chen, Zhenyang Zhang, Zhen Wang, Qishan Wang, Zhe Zhang, Yuchun Pan

**Affiliations:** 1 College of Animal Sciences, Zhejiang University, Hangzhou, 310058 Zhejiang China; 2 Hainan Institute of Zhejiang University, Building 11, Yongyou Industrial Park, Yazhou Bay Science and Technology City, Yazhou District, Sanya, 572025 Hainan China

## Abstract

**Background:**

Natural and artificial selection for more than 9000 years have led to a variety of domestic pig breeds. Accurate identification of pig breeds is important for breed conservation, sustainable breeding, pork traceability, and local resource registration.

**Results:**

We evaluated the performance of four selectors and six classifiers for breed identification using a wide range of pig breeds (N = 91). The internal cross-validation and external independent testing showed that partial least squares regression (PLSR) was the most effective selector and partial least squares-discriminant analysis (PLS-DA) was the most powerful classifier for breed identification among many breeds. Five-fold cross-validation indicated that using PLSR as the selector and PLS-DA as the classifier to discriminate 91 pig breeds yielded 98.4% accuracy with only 3K single nucleotide polymorphisms (SNPs). We also constructed a reference dataset with 124 pig breeds and used it to develop the web tool iDIGs (http://alphaindex.zju.edu.cn/iDIGs_en/) as a comprehensive application for global pig breed identification. iDIGs allows users to (1) identify pig breeds without a reference population and (2) design small panels to discriminate several specific pig breeds.

**Conclusions:**

In this study, we proved that breed identification among a wide range of pig breeds is feasible and we developed a web tool for such pig breed identification.

**Supplementary Information:**

The online version contains supplementary material available at 10.1186/s12711-023-00788-0.

## Background

Pigs are one of the main sources of animal protein for humans and are commonly used for biomedical research. Over the past 9000 years, various pig breeds have been produced through natural and artificial selection [1]. According to a recent report of the Food and Agriculture Organization of the United Nations, there are currently approximately 600 pig breeds worldwide, most of which are found in Asia and Europe [2]. Breed identification of pigs is crucial for breed conservation, sustainable breeding, pork traceability, and local resource registration. Local pig breeds represent an important genetic resource with considerable genetic variability, however, most of these breeds are at risk of extinction because of a multitude of challenges, including emerging diseases, climate change, and competition from international commercial breeds [3]. Accurate breed identification is the premise of undertaking measures to alleviate such trends. Furthermore, the increasingly abundant whole-genome sequence (WGS) and single nucleotide polymorphism (SNP) chip datasets that are available in curated databases represent important resources to characterize the genetic diversity of pigs [4].

The long history of pig domestication has shaped the genetic structure of pig breeds, which facilitates breed identification based on genetic information. Molecular genetic markers, such as short tandem repeats (STRs) and SNPs, can be used to identify genetic heterogeneity among breeds. For more than 30 years, the highly variable STRs have been used for breed identification in various species [5]. However, due to advances in high-throughput sequencing and genotyping technology, large numbers of SNPs have now been characterized and these have replaced STRs in animal genetic research over the past decade [6]. Heterogeneous SNPs are referred to as breed informative markers and they are used for breed identification and assessing genetic breed composition and signatures of selection. Commercial SNP chips for pigs have mainly been developed based on the most heterogeneous SNPs between a wild boar population and four cosmopolitan breeds [7] but, to discriminate breeds of interest, it is necessary to select a more specific subset of these SNPs. Compared to commercial SNP chips, selection of breed-specific informative SNPs can remove noisy and redundant SNPs, and can reduce the cost of breed identification.

Current studies on breed identification typically include two steps, i.e., (1) selecting breed informative SNPs and (2) fitting a classification model using these SNPs [8, 9]. In machine-learning methods, a ‘selector’ is defined as any approach that selects a subset of features from original features by removing those that are irrelevant and noisy, while a ‘classifier’ is defined as any algorithm that assigns test individuals to labeled classes [10, 11]. Some machine-learning approaches, however, accomplish both of these simultaneously. For example, random forest (RF) produces results for both dependent variable significance and classification [12]. Accordingly, in this study, the approaches used for the selection of breed informative SNPs are referred to as selectors, and models used for classification analysis are referred to as classifiers.

For selection of breed informative markers, commonly used methods are the delta method, Wright’s fixation index (F_ST_), and principal component analysis (PCA) [6]. For breed assignment analysis, various machine-learning methods have been used, including RF [13, 14], support vector machine (SVM) [8], and k-nearest neighbor (KNN) methods [9], with performance differing only slightly between classifiers [9]. Most studies that have compared different selectors and classifiers for breed identification of farm animals have, however, included at most 20 specific breeds. For example, a panel containing 64 breed informative markers was developed to differentiate Iberian and Duroc pigs [7] and a panel of 20 breed informative markers was selected using F_ST_ to discriminate 13 pig breeds using different machine-learning methods [9]. These studies often provide a protocol to design a panel for breed identification of specific breeds. However, breed informative markers for a limited number of breeds cannot be applied to other breeds. Consequently, users need to develop a new panel for breed identification of the breeds that they are interested in, which requires the construction of a specific reference population, alongside the selection of breed informative markers based on this reference population. Thus, a publicly available reference database that includes many breeds can obviate the need for these time-consuming and costly tasks.

As a result of the massive application of SNPs in genomic selection and genome-wide association analysis in pigs, abundant SNP information is now publicly available from commercial SNP chips and WGS datasets for many pig breeds. These data can help develop a public database for the identification of breeds from across the globe. However, the performance of breed identification methods remains unclear when the number of breeds is very large. In addition, while it is well known that more SNPs are required to distinguish among a large number of breeds, the minimum number of SNPs to distinguish pig breeds globally is unknown.

In this study, we curated data from three commercial SNP chips and a WGS dataset that represent 3605 individuals from various pig breeds worldwide. To confirm the applicability of breed identification in a wide range of pig breeds, we compared the performance of four selectors and six classifiers using internal cross-validation or an external independent dataset. We generated a reference dataset for breed identification and developed an interactive web tool, iDIGs, to perform pig breed identification by simply uploading SNP information of testing individuals. The iDIGs tool also allows users to design panels of breed informative markers for specific pig breeds without requiring additional pre-prepared data.

## Methods

**Fig. 1:**
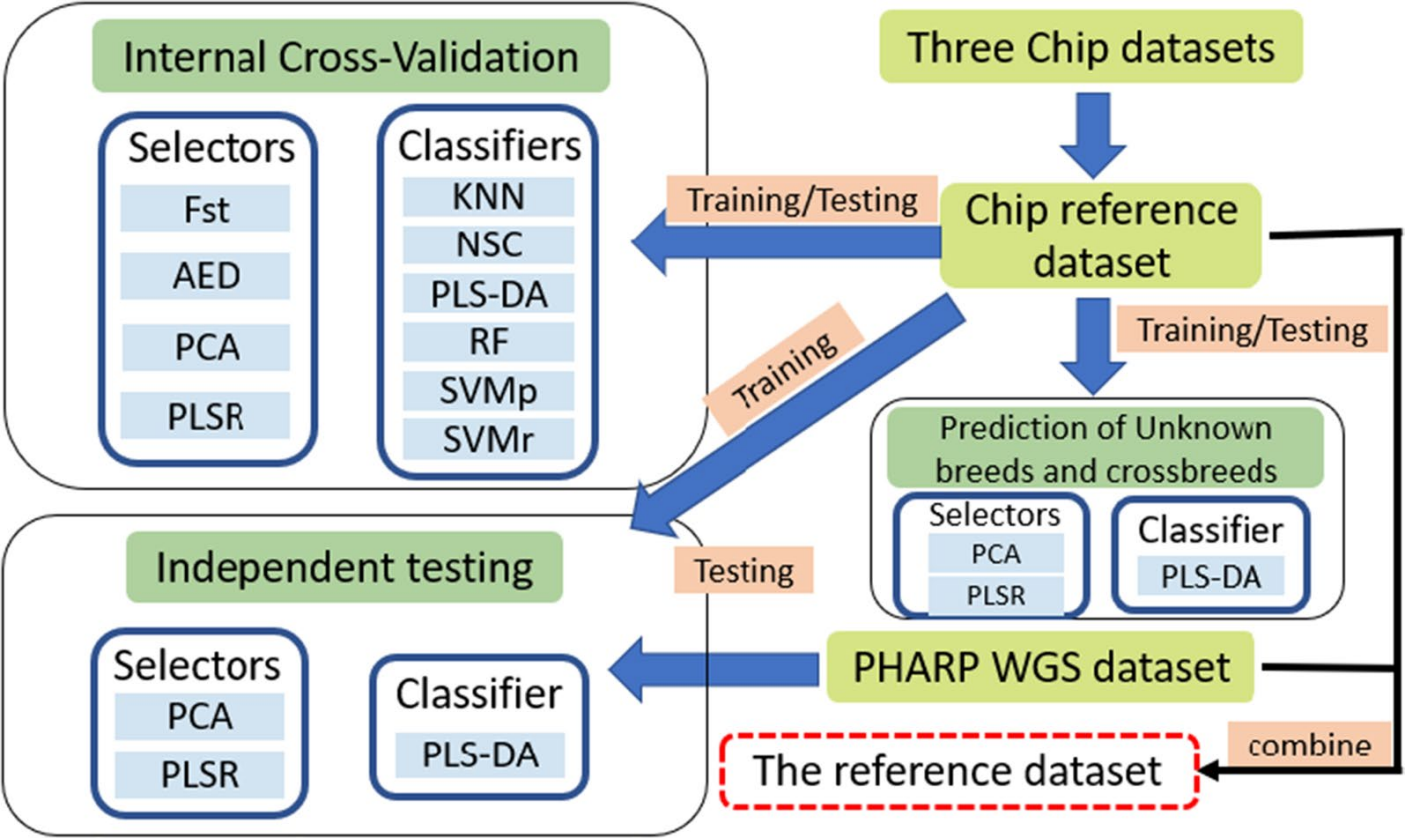
Flowchart of the evaluation of model performance and construction of the reference database. AED: average Euclidean distance; F_ST_: fixation index; PCA: principal component analysis; PLSR: partial least squares regression; KNN: k-nearest neighbor; NSC: nearest shrunken centroids; PLS-DA: partial least squares-discriminant analysis; RF: random forest; SVMp: support vector machine with polynomial kernel; SVMr: support vector machine with Gaussian radial basis function

A flowchart that depicts the general scheme of this study is in Fig. 1.

### Data collection and pre-processing

Three pig SNP chip datasets were downloaded from the Dryad Digital Repository (https://datadryad.org/) [15] and the Figshare database (https://figshare.com/) [16, 17]. Breeds with less than ten individuals were removed. Table 1 shows a summary of these datasets. The three datasets were merged and SNPs with physical locations on the sex chromosomes and with minor allele frequencies lower than 0.01 were removed. The software Beagle (v5.2) [18] was used to impute sparse missing genotypes using default parameters. Crossbreds were removed and served as an independent testing set in downstream analyses. The admixture (v1.3.0) software [19] was used to investigate the genetic structure of all animals included in the chip reference dataset. Animals with admixture profiles that differed markedly from those of other animals from the same breed were removed and the remaining samples were considered purebred. Since the downloaded raw data were based on the 10.2 *Sus scrofa* assembly, we also generated a chip reference dataset based on the 11.1 *Sus scrofa* assembly [20]. A WGS dataset from the Pig Haplotype Reference Panel (PHARP) [21] was also included in this analysis. PHARP is a free genotype imputation service that comprises various pig breeds from across the globe. SNPs in the PHARP data were generated using the GATK pipeline [22] with the 11.1 *Sus scrofa* assembly as the reference genome.

**Table 1:** Summary of the datasets used

Dataset	Sample size	Number of breeds or populations	Number of SNPs	References
Chip dataset 1	2113	146	47,700	[23]
Chip dataset 2	263	10	32,012	[16]
Chip dataset 3	124	4	23,532	[24]
PHARP v2	1585	99	34,265,424	[21]

### Selection of breed informative SNPs

To compare the performance of different selectors, average Euclidean distance (AED), F_ST_, PCA, and partial least squares regression (PLSR) were used to select breed informative SNPs. These four selectors each assign a different statistic to each SNP that reflects the heterogeneity of that SNP between breeds. The four statistics were calculated as follows:

- AED: the AED value for a certain SNP across all breeds was calculated as:
  1 $$\mathrm{AED}= \frac{1}{{\mathrm{C}}_{T}^{2}}\sqrt{\sum \limits_{i=1}^{T} \sum \limits_{j>i}^{T}{\left({f}_{i}-{f}_{j}\right)}^{2},}$$
  where $T$ is the number of breeds, ${f}_{i}$ and ${f}_{j}$ are the allele frequencies for the SNP in breeds $i$ and $j$, respectively, and ${\mathrm{C}}_{T}^{2}$ is the number of combinations that select two breeds from the $T$ breeds.
- F_ST_: estimates of Weir and Cockerham’s global F_ST_ [25] were calculated using the PLINK (v1.9) [26] software.
- PCA: first, the allele frequencies of each SNP in each breed were calculated and a breed-specific allele frequency matrix was generated. Then, PCA was performed on the matrix of breed-specific allele frequencies using the *prcomp* function of the R software [27]. The loading of a SNP was defined as the sum of the squares of the first ten eigenvectors, which were ordered by their corresponding eigenvalues.
- PLSR: PLSR is a supervised learning algorithm that reduces the explanatory variables to a smaller set of uncorrelated components and performs a regression on these components. This is appropriate for a regression analysis for which multicollinearity of explanatory variables is high. Several studies have shown the efficiency of PLSR for the selection of breed informative SNPs [7, 8]. Considering the large number of SNPs used in this study, we applied 150 components in the PLSR using the R software package pls [28]. The sum of the square of the regression coefficients for each SNP calculated by PLSR was used to select breed informative SNPs.

Using these four statistics, nine SNP panels with different densities (200, 500, 800, 1K, 3K, 5K, 7K, 10K, and 12K) based on their magnitude were constructed to assess the impact of different marker densities.

### Classifiers for pig breed identification

Five multi-class classification methods were used to perform breed assignment using different numbers of breed informative SNPs, including KNN, nearest shrunken centroids (NSC), partial least squares discriminant analysis (PLS-DA), RF, SVM with a polynomial kernel (SVMp), and SVM with a Gaussian radial basis function (SVMr). The model parameters for these six classifiers are summarized in Table 2. These methods are described in the following paragraphs:

- KNN: KNN is a non-parametric algorithm for multi-class classification. For each test individual (the individual whose breed needs to be predicted), it calculates its distance to all the training individuals (the individuals whose breed is known) and chooses the first k nearest training individuals. Then, it assigns the test individual with the mode of the breed labels of the k nearest training individuals. KNN classification was performed using the R package class [29]. The parameter k was set to 5 and Euclidean distances were used to calculate the distances between training and test individuals.
- NSC: NSC classification was first introduced for the diagnosis of multiple cancer types based on DNA microarrays [30], using data with a large number of features and a relatively small number of individuals. Thus, NSC was considered suitable for performing breed assignment analysis using SNP data. NSC obtains centroids for all breeds by averaging each SNP in each breed and then standardizing centroids using the within-breed standard deviation of all SNPs. After standardization, NSC shrinks these centroids towards the overall centroid, which is calculated by averaging each SNP for all breeds. The predicted class of a test individual is the class of its nearest centroid. NSC classification was performed using the R package caret with default parameters [31].
- PLS-DA: PLS-DA is a discrimination method based on PLSR and recently has become increasingly popular in the research area of metabolomics for the purpose of classifying individuals into either a case or control group based on their metabolic profile [32]. As a variant of PCA in supervised learning, PLS-DA is capable of feature selection as well as classification [33]. For the PLS-DA analysis, PLSR was used to model the relationship between the breed labels and the first 150 uncorrelated components of the SNP genotype matrix. Prediction values of all breeds from PLSR were then converted to a probability distribution of all breed labels using the softmax function [31]. The breed label with the highest probability was considered to be the predicted label. PLS-DA classification was performed using the R package caret [31].
- RF: RF is an ensemble learning method that assembles results from several decision trees to avoid overfitting by a single decision tree model. For classification jobs, the output of the RF model is the mode of the predicted class from all decision trees. RF models were previously considered adequate for the selection of breed informative markers [34] and for breed classification [13, 14]. RF modeling was performed using the R package randomForest with default parameters [35].
- SVMp and SVMr: SVM is a well-known and fast machine-learning algorithm that can be used for regression and classification [36]. SVM uses different kernel functions to map the raw input to high-dimensional feature spaces and then finds a maximum-margin hyperplane that can separate the data into classes in high-dimensional spaces [37]. SVM is an efficient classifier, especially when dealing with nonlinear separable problems. For multi-class classification problems, SVM adopts a one-to-one approach, which breaks the multiclass problem into multiple binary classification problems. SVMp uses a polynomial kernel to map the input SNP matrix to high-dimensional feature spaces, whereas SVMr uses the Gaussian radial basis function [38]. SVMp and SVMr were performed using the R package e1071 with default parameters [38].

**Table 2:** Parameters used in the classifiers

Classifier	Tuned parameters	Default parameters
KNN	K = 3	l = 0
NSC	–	threshold = 30
PLS-DA	ncomp = 150	probMethod = "softmax"
RF	–	ntree = 500, mtry = sqrt(p),
SVMp	–	degree = 3, scale = true, gamma = 1/p, cost = 1
SVMr	–	scale = true, gamma = 1/p, cost = 1

p is the number of SNPs

### Evaluation of model performance using the chip reference dataset

Stratified five-fold cross-validation was used to evaluate the performance of all classification methods with the chip reference dataset by partitioning this dataset into five equally-sized subsets of individuals, while maintaining each subset to have roughly equal proportions of all breeds. To obtain reliable estimates of model performance, we performed the stratified five-fold cross-validation five times, and the average accuracy across the five times five-fold cross-validation was used as a measurement of model performance.

### Prediction of unknown breeds and crossbreds

We randomly selected eight breeds from the chip reference dataset as unknown breeds. All individuals that belonged to these unknown breeds were extracted as testing sets. For the other breeds in the chip reference dataset, we randomly extracted one third of the individuals for testing. Individuals from two crossbreds, CSLM (LargeWhite $\times$ Meishan) and CSPL (Pietrain $\times$ LargeWhite), from the chip dataset 1 were also extracted as testing sets, which were removed when constructing the chip reference dataset. Information on the testing sets is in Table 3.

**Table 3:** Summary of unknown breeds and crossbreds

Breeds	Abbreviation	Sample size	Type
American Feral	AMFE	36	Unknown breeds
Berkshire	BK	38	Unknown breeds
CostaRica Creole	CRCR	12	Unknown breeds
DeBao	DB	15	Unknown breeds
HeTaoDaEr	HTDE	16	Unknown breeds
ShaZiLing	SZL	11	Unknown breeds
Ukrainian Pork Swine	UAPS	12	Unknown breeds
YueDongHei	YDH	25	Unknown breeds
LargeWhite × Meishan	CSLM	20	Crossbreds
Pietrain × LargeWhite	CSPL	20	Crossbreds

The PCA and PLSR methods were used to select three panel densities (1K, 5K, and 10K) because of their high performance in cross-validation. PLS-DA was used to predict breed labels of test individuals with different panel densities. The regression coefficients for each breed label from PLS-DA were transformed to a probability distribution using the softmax function. In the cross-validation analyses, the breed label with the highest probability was set as the predicted label. A predefined threshold was used to identify unknown breeds and crossbreds. When the highest probability was lower than the threshold, the predicted label was set as “unknown”. To identify an adequate threshold, we tested different thresholds from 0.01 to 0.03 with increments of 0.001.

### Evaluation of model performance using the independent WGS dataset

Individuals whose breed labels occurred in the chip reference dataset were extracted from the WGS PHARP datasets. In total, 26,858 SNPs that were shared between the chip reference (version 11.1) and the WGS PHARP datasets were used. PCA and PLSR were chosen to select different panel densities because of their performance in cross-validation. PLS-DA was used to predict breed labels of individuals in the independent testing dataset with different panel densities, ranging from 1000 to 15,000, with increments of 1000.

### Construction of the reference dataset and design of the iDIGs webtool

Breeds from PHARP with more than 10 individuals were merged with the chip dataset to produce a larger reference dataset. To generate a high-quality SNP reference dataset for use with different genotyping platforms, SNPs that overlapped between the chip and the PHARP reference dataset were retained and imputed. The R package Shiny [39] was used to construct the web interface for breed identification based on the reference dataset.

## Results

### Data and pre-processing

After basic quality control of the merged dataset, we obtained genotypes on 2333 individuals and 46,974 common SNPs in the chip dataset. Forty crossbreds were removed and an additional 21 individuals were removed due to their inconsistent admixture profile (see Additional file 1: Fig. S1). In total, genotypes on 2272 individuals and 46,974 common SNPs were retained in the chip dataset. The conversion from the 10.2 to the 11.1 *Sus scrofa* assembly resulted in the loss of 1231 SNPs. Therefore, the chip reference dataset (*Sus scrofa* 11.1) contained genotypes on 2272 individuals and 45,743 SNPs and comprised 91 breeds, of which 41 were Chinese indigenous breeds. Breeds used in the chip reference dataset and the number of individuals for each breed are in Additional file 2: Table S1.

### Performance evaluation of classifiers in the chip reference dataset

**Fig. 2:**
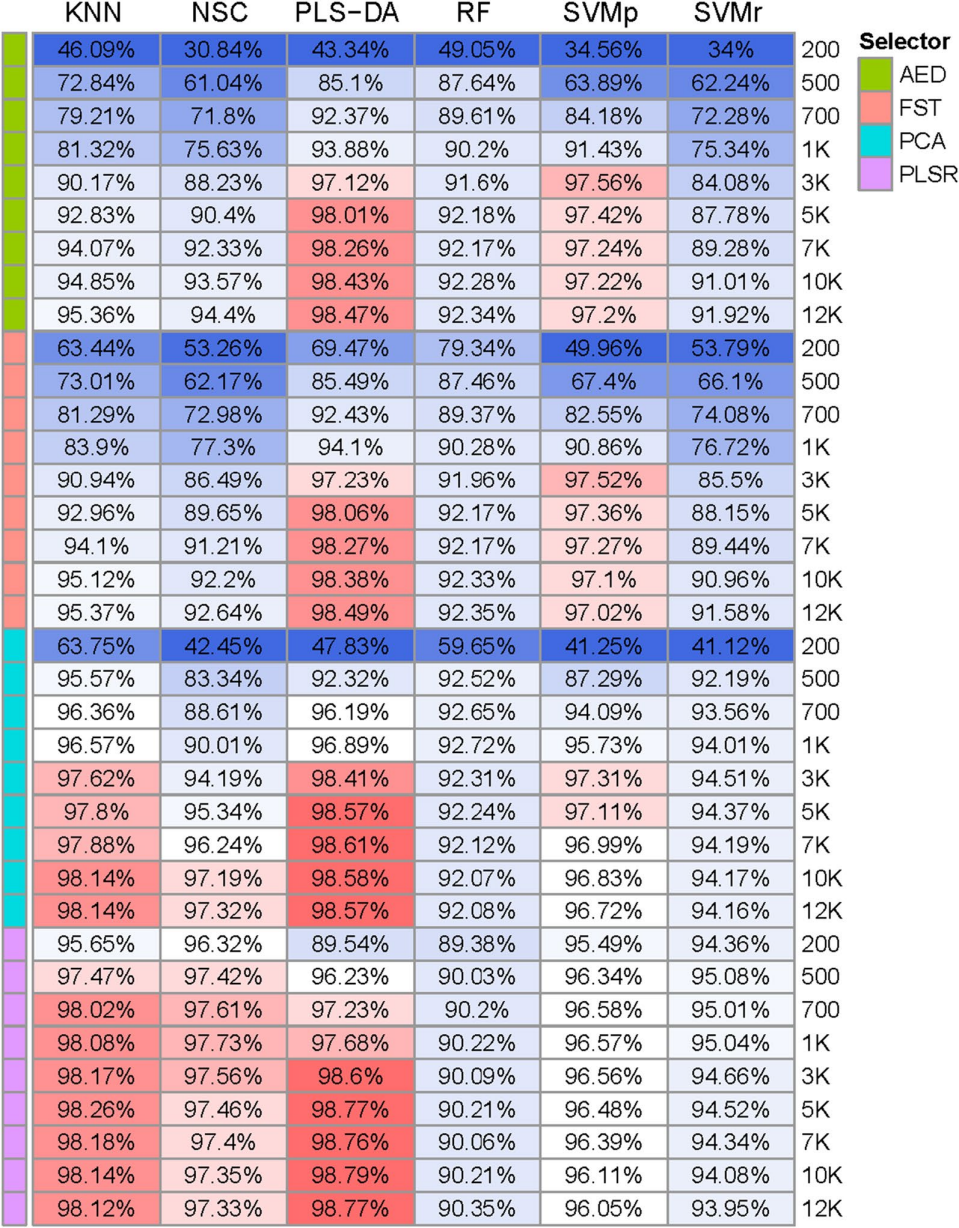
Average accuracy of breed identification in the chip reference dataset. The average identification accuracy of five times five-fold cross-validation in merged chip data using 36 SNP panels and six classifiers. The 36 SNP panels were constructed using four models (EDA, F_ST_, PCA, and PLSR) and nine SNP densities (200, 500, 800, 1K, 3K, 5K, 7K, 10K, and 12K)

We performed a stratified five-fold cross-validation five times for each classifier and each SNP panel using the chip reference dataset. The average accuracy of different classifiers is shown in Fig. 2. In general, the average accuracy increased as the number of markers increased for all classifiers. Using PLS-DA as a classifier with the 10K SNP panel resulted in the highest accuracy (98.8%). In terms of selectors, PCA and PLSR exhibited significantly higher efficiencies than AED and F_ST_. PLSR was the most efficient marker selection method, especially when the number of markers was small. When using PLSR to select breed informative markers, almost all classifiers obtained an average accuracy greater than 90%, although only 200 markers were selected. When using the PLSR selector, the 1K and 10K marker densities led to similar predictive accuracies. In general, PLS-DA was the best classifier. Average accuracies were less than 98.3% for of all methods, except for PLS-DA.

### Prediction of unknown breeds and crossbreds

**Fig. 3:**
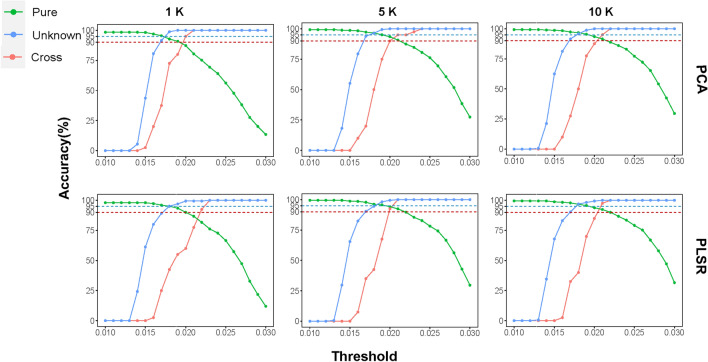
Prediction accuracies of pure breeds, unknown breeds, and crossbreds using six SNP panels. The six SNP panels were constructed using two models (PCA and PLSR) and three SNP densities (1K, 5K, and 10K)

In total, 756 individuals, including 551 pure individuals, 165 unknown individuals, and 40 cross individuals, were used to establish an adequate threshold for breed identification when considering the presence of unknown breeds or crossbreds (see Additional file 3: Table S2). We evaluated the prediction accuracy for purebreds, unknown breeds, and crossbreds independently, and we attempted to identify an adequate threshold to balance the trade-off between pure breed identification and incorrect detection. For the unknown breeds and crossbreds, an “unknown” identification was considered to be a correct outcome. The prediction accuracy under different thresholds are shown in Fig. 3. As expected, the prediction accuracy of unknown breeds and crossbreds increased and that of pure breeds decreased with increasing thresholds. When using 1K SNPs, it was not possible to simultaneously obtain accuracies higher than 90% for all three categories, regardless of the threshold used. The utilization of 5K or 10K SNPs results in an almost equivalent accuracy of prediction for all three types of individuals by both selectors. The best trade-off between prediction accuracy of pure breeds and the other two categories occurred when using 5K SNPs, PLSR as the selector and a threshold of 0.021, as it resulted in accuracies of 92.4% for purebreds and 100% for unknown breeds and crossbreds. In almost all situations, a threshold of either 0.02 or 0.021 resulted in a satisfactory trade-off, where the prediction accuracy for all three categories surpassed 90%.

### Independent evaluation of model performance using the WGS dataset

**Fig. 4:**
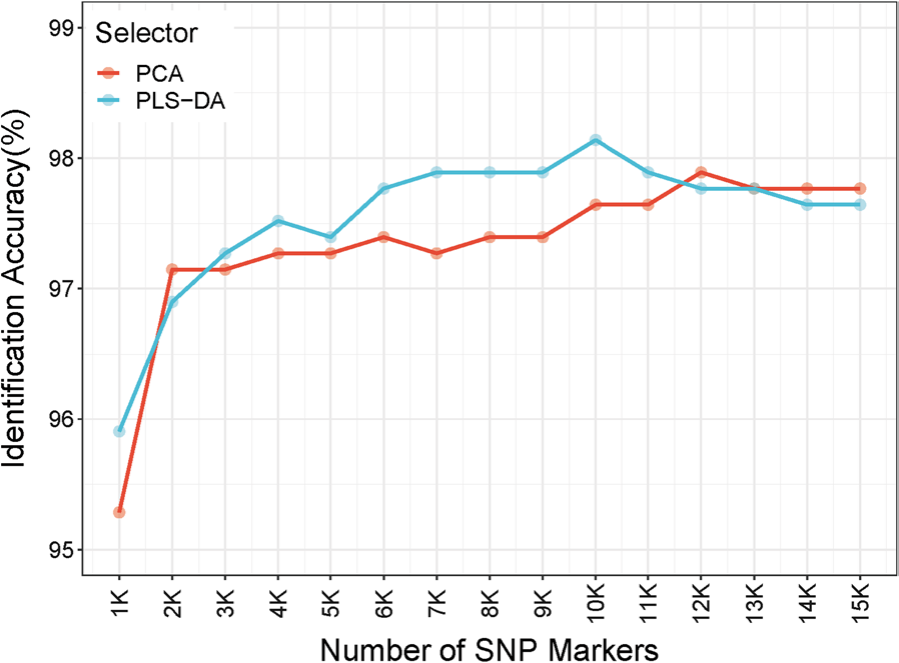
Identification accuracy in the independent WGS dataset. Identification accuracy of the independent WGS dataset using different panels selected by PCA and PLS-DA. Thirty different panels were constructed using two selectors (PCA and PLSR) and 15 SNP densities

Data on 806 individuals from 48 breeds were used as an independent WGS dataset (see Additional file 4: Table S3). We used PLSR and PCA to select 15 SNP panels with densities ranging from 1K to 15K, and then used PLS-DA to assign a breed label to each individual in the WGS dataset for different panels (Fig. 4). Overall, the prediction accuracy of these two selectors increased as the number of markers increased. With more than 4K markers, both selectors reached a stable prediction accuracy of approximately 97.8%. The minimum accuracy of 95.9% was obtained with the 1K panel selected by PLSR, while the maximum accuracy of 97.9% was obtained for several panels. The accuracy obtained with PLSR was almost always slightly higher or the same as the accuracy obtained with PCA when the number of markers exceeded 2.5K. The incorrectly assigned individuals from the WGS dataset tended to be consistent across different marker densities, indicating the underlying genetic distance between the same breeds in the chip reference dataset and the WGS dataset leads to inaccurate prediction. These incorrectly assigned individuals were discarded and the two types of datasets were then merged.

### Construction of the reference dataset and design of the iDIGs webtool

**Fig. 5:**
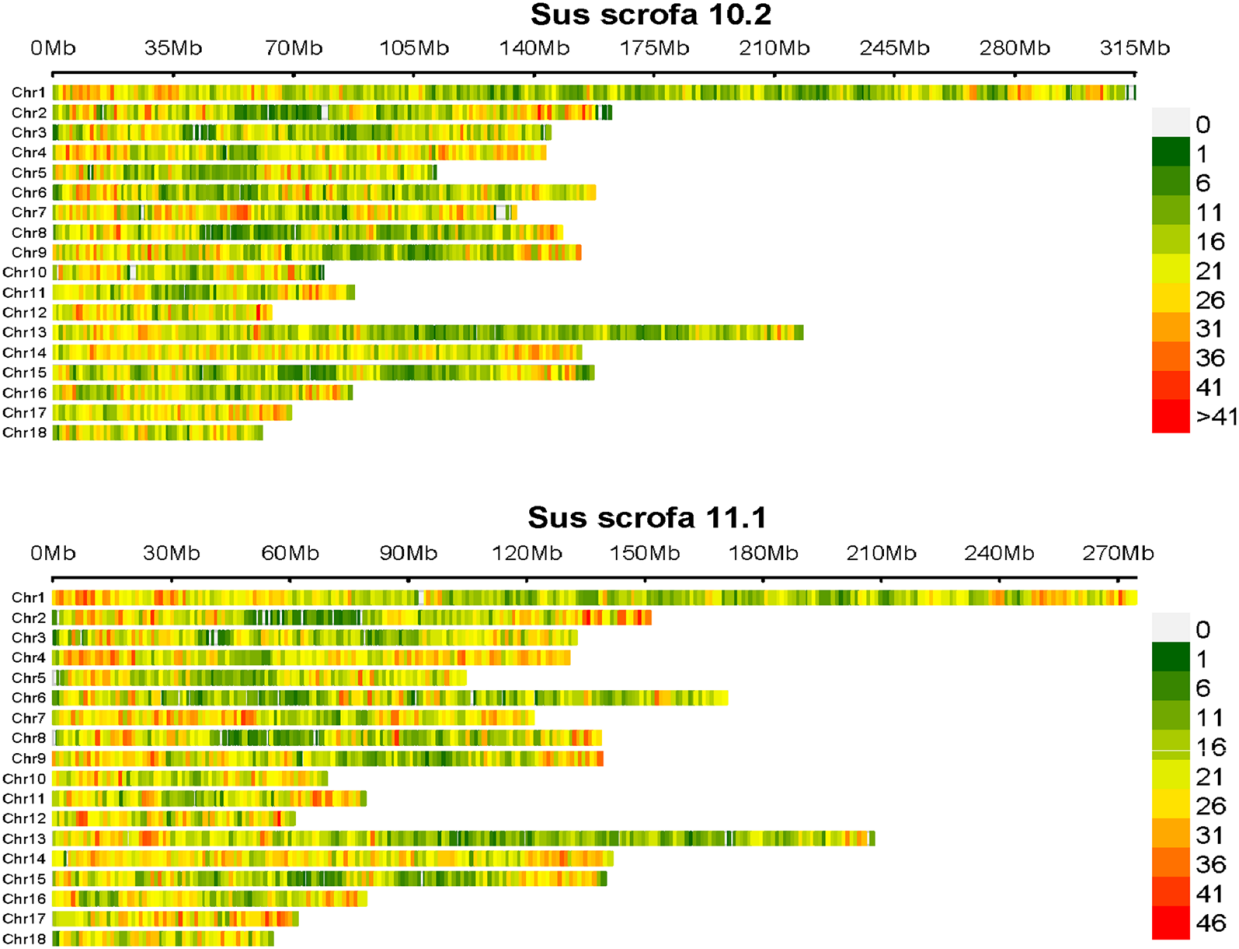
Genome distribution of SNPs in the reference dataset. Genome distribution of 45,743 SNPs in the reference dataset in 10.2 and 11.1 *Sus scrofa* assemblies

By combining the chip reference dataset and the PHARP datasets after removing the incorrectly assigned individuals (see above), we produced a reference dataset with 45,743 SNPs and 3605 individuals from 124 pig breeds. The information included for each breed is in Additional file 5: Table S4. These 45,743 SNPs covered almost all the genomic regions (Fig. 5).

Based on the reference dataset, the web tool (iDIGs) was designed for pig breed identification-related analyses, including pig breed identification and design of SNP panels (breed informative marker selection) for the identification of specific pig breeds. The workflow of breed identification analysis is as follows. First, the PLINK binary file needs to be uploaded to the iDIGs web server and the version of the reference genome should be chosen. iDIGs renames the SNP ID to “Chr:Pos:allele1:allele2”, where “Chr” and “Pos” are the chromosome and position localizations of this SNP, respectively; “allele1” and “allele2” are the alleles that have the smaller and larger ASCII code, respectively. Next, iDIGs extracts the common SNPs between the uploaded data and the reference data, based on SNP ID. Then, SNPs in the uploaded data are recoded as the number of reference alleles. The reference allele of each SNP in the uploaded data is consistent with the reference allele used in the reference dataset. By default, iDIGs selects 5000 breed informative SNPs using PLSR. If the number of common SNPs is smaller than 5000, the marker selection procedure is skipped. iDIGs then retrains the PLS-DA model using the selected SNPs and assigns a breed label for each sample in the uploaded file.

In order to design a SNP panel for the identification of specific breeds, no additional files need to be uploaded. Users need to choose the panel density and input the breeds that are targeted to be differentiated from the available 124 breeds. Here, users can input multiple breed IDs or a single breed ID. If multiple breed IDs are used, markers that are most discriminating among all input breeds are selected. When using a single breed ID, markers that can distinguish the breed from all other breeds in the reference database are selected. After obtaining the panel of breed informative markers, iDIGs automatically conducts five-fold cross-validation to evaluate the performance of the panel. Users can then judge if the number of SNPs in the panel is sufficiently large for identification of the breeds of interest.

## Discussion

### Advantages and disadvantages of iDIGs

In this study, we generated a reference dataset with 124 pig breeds and built the web tool iDIGs for pig breed identification using this reference dataset. To our knowledge, this is the first breed identification study based on worldwide pig breeds, as well as the first public tool for the identification of a wide range of pig breeds. In addition to breed identification, iDIGs can also be used to authenticate the breed label of publicly available data. It is acknowledged that individuals within such datasets may possess incorrect or inaccurate breed labels, which can result in erroneous conclusions if used in genomic analyses (e.g. selection signatures). As such, it is recommended that breed labels be verified using iDIGs prior to conducting any related analyses.

While previous studies on breed identification only selected tens or hundreds of markers [7, 34, 40], in the current study, 3K markers were the minimum number required to obtain 98% accuracy. The number of markers required for breed identification depends on the number of breeds being considered [41]. Previous studies have primarily focused on a limited number of specific breeds and were able to achieve acceptable results with a small SNP panel. However, the design of iDIGs, which is intended to handle a wide range of pig breeds, highlights the inefficiency of such small SNP panels. The minimum number of SNPs required is mainly determined by the heterogeneity of SNPs across all breeds. By default, iDIGs assigns a test individual to one of the 124 possible breeds, thus requiring a greater number of SNPs to accurately discriminate against each breed. Our reference dataset was generated using commercial SNP chips and comprises over 40,000 SNPs, ensuring that the number of common SNPs is not a concern for commercial chip or WGS data. For reduced-representation sequencing, users can first perform imputation using a public imputation database, such as PHARP. In addition, iDIGs allows users to select a subset of breeds from the 124 reference breeds for breed identification, which can help reduce the minimum number of required SNPs and improve prediction accuracy.

”Breed” is a dynamic concept and the genetic characteristics of a breed may change over time as a result of selection, genetic drift, and other gene flow events. Therefore, the increasing genetic distance of the test individuals from individuals from the same breed in the reference dataset will decrease prediction accuracy. This can be addressed by continuously updating the reference dataset.

### Prediction of unknown breeds

In iDIGs, each test individual is assigned to one of 124 breed labels, even if the true breed label is not included in our reference dataset. It was, however, not possible to include all worldwide pig breeds in our reference dataset. To overcome this shortcoming, we predefined a threshold for the model fitness of PLS-DA and reset the predicted breed as “unknown” if the probability of the predicted breed is smaller than this threshold. iDIGs uses 0.02 as the default threshold, but users can change this parameter to achieve a better trade-off between true positive and false positive rates. If the threshold is set to “NA”, iDIGs disables the function of unknown breed prediction.

### Designing a small SNP panel for pig breed identification

iDIGs also provides a function to select breed informative markers to differentiate among specific breeds. All SNPs in the reference dataset were derived from commercial SNP chips and are, therefore, easy to design and to genotype for a small panel. Building a reference dataset is the prerequisite for the design of a panel for breed identification but this is time-consuming and expensive. With iDIGs, users can directly use the reference database to select breed informative markers to design panels for most situations because it includes 124 pig breeds that were distributed across 47 sites in 24 countries. However, it is important to consider that the performance of the panels generated by iDIGs may be subject to bias due to the limited number of individuals representing certain breeds in the reference dataset. Therefore, to mitigate this potential bias, we suggest increasing the size of the SNP panel.

### Analysis of genomic breed composition

With economic globalization, gene flow between local and foreign breeds will increase, which may threaten the purity and integrity of local breeds. Some local breeds may be crossed with commercial breeds in order to accelerate lean growth and improve carcass mass, which may disintegrate precious local genetic resources. Analysis of breed purity is referred to as genomic breed composition (GBC) analysis [42, 43]. GBC reflects the genomic contribution of each ancestral breed to the genome of the test animal. Multiple methods can be used to estimate the GBC of crossbreds, such as linear models, supervised admixture models [44], and Bayesian inference of breed composition (BIBI) methods [45]. He et al. [41] reported high correlations between GBC that were calculated from a linear model and those from an admixture model. Almost all the GBC methods developed to date require all potential ancestral breeds to be included in the reference database.

Breed identification is a qualitative analysis, while GBC analysis is quantitative, which makes it more complex and requires detailed prior knowledge (all potential ancestral breeds) of candidate individuals. Because of this, iDIGs currently does not include a function for GBC analysis with the linear model. In addition, because GBC analyses are based on allele frequencies of breeds, sample sizes in our reference database are too small for some breeds to calculate accurate allele frequencies. In addition, because natural and artificial selection can result in multiple strains of a given breed, allele frequencies calculated from individuals in public databases may be less representative for some populations. We did attempt GBC analyses for some crossbreds, but obtained no reliable results. Since we are not sure about the true pedigree of the crossbreds (which were downloaded from public databases), we did not include these results here. To eliminate the bias introduced by public data, we designed an R package (https://github.com/JanMiao/GBC) to compile reference data from user-provided data. The procedure for the construction of the reference database includes removing outliers, removing relatives, and selection of breed informative markers. This also allows users to perform GBC analysis with multiple algorithms based on their own reference data.

## Conclusions

In this study, we first proved that breed identification for a wide range of breeds is feasible. We constructed a reference dataset for breed identification using multiple datasets and developed a web tool (iDIGs) based on the reference database. iDIGs can be used to identify breed identity and to design small panels for the identification of specific pig breeds without the need to prepare a separate reference population.

## Supplementary Information

**Additional file 1: Figure S1.** Plots of admixture profiles before and after removing outliers; (a) shows the admixture profile of raw reference individuals, and (b) shows the admixture profile of the remaining individuals after removing 21 outliers.

**Additional file 2: Table S1.** Information on the breeds included in the chip reference dataset.

**Additional file 3: Table S2.** Information on the individuals used as crossbreds, unknown breeds, and testing pure breeds.

**Additional file 4: Table S3.** Information on the breeds used as independent testing dataset.

**Additional file 5: Table S4.** Information on the breeds used in the reference dataset.

## Data Availability

Three publicly available Chip datasets can be downloaded from the Dryad Digital Repository (http://dx.doi.org/10.5061/dryad.30tk6) and Figshare database (https://figshare.com/s/459f0a85cba4f694d8f8 and https://doi.org/10.6084/m9.figshare.7588235.v1). The PHARP WGS dataset can be obtained upon request. Scripts used in this study are available from https://github.com/JanMiao/iDIGs.
