# Supplementary figures and images for: A web tool for the global identification of pig breeds
Source: Genet Sel Evol. 2023 Mar 21;55:18. doi: 10.1186/s12711-023-00788-0 (PMC10029154; doi:10.1186/s12711-023-00788-0)

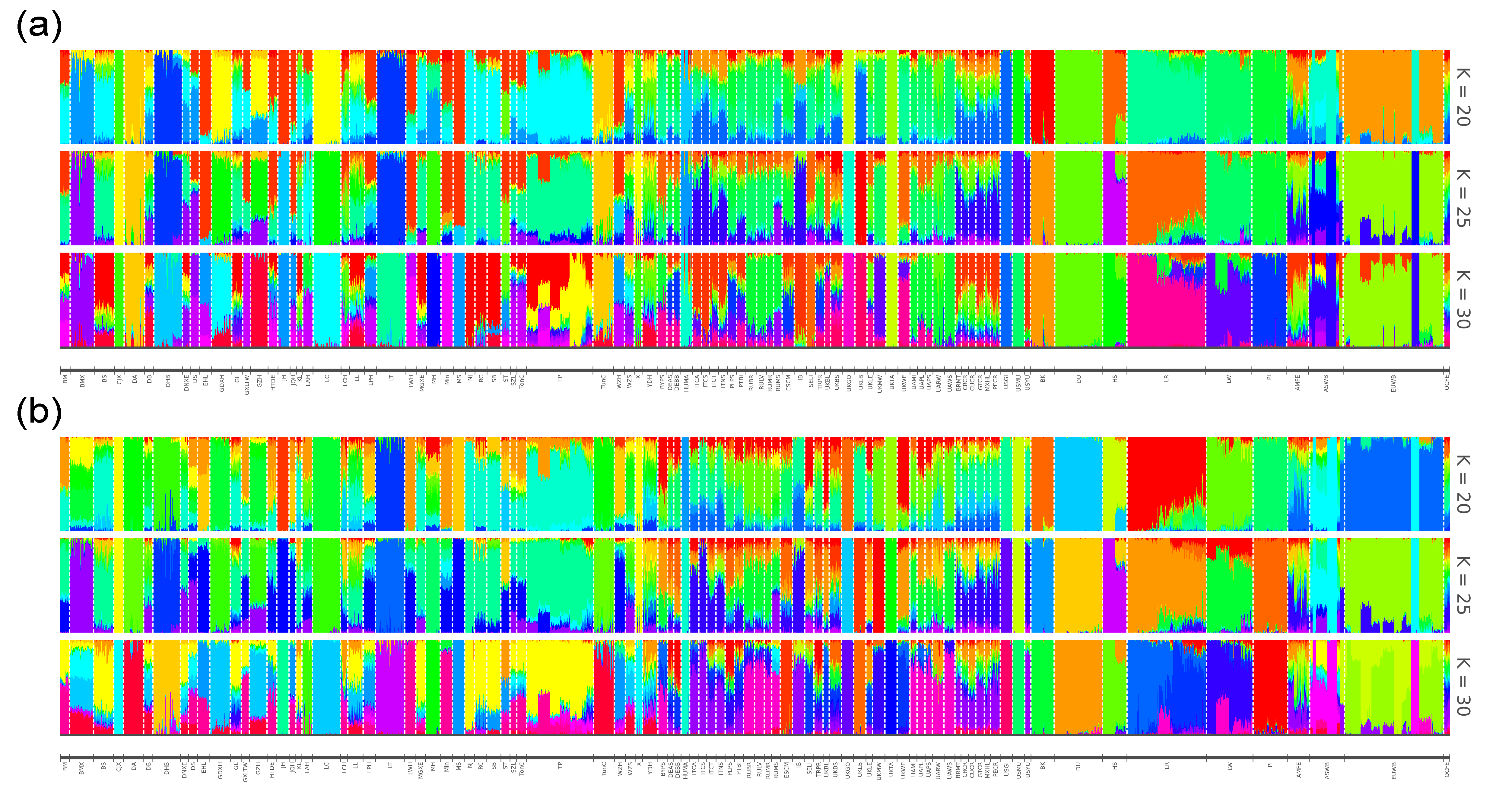

Supplement: Supplementary file 1 — Additional file 1: Figure S1. Plots of admixture profiles before and after removing outliers; (a) shows the admixture profile of raw reference individuals, and (b) shows the admixture profile of the remaining individuals after removing 21 outliers. [file 12711_2023_788_MOESM1_ESM.png]
